# *De novo* whole-genome assembly in *Chrysanthemum seticuspe*, a model species of Chrysanthemums, and its application to genetic and gene discovery analysis

**DOI:** 10.1093/dnares/dsy048

**Published:** 2019-01-27

**Authors:** Hideki Hirakawa, Katsuhiko Sumitomo, Tamotsu Hisamatsu, Soichiro Nagano, Kenta Shirasawa, Yohei Higuchi, Makoto Kusaba, Masaji Koshioka, Yoshihiro Nakano, Masafumi Yagi, Hiroyasu Yamaguchi, Kenji Taniguchi, Michiharu Nakano, Sachiko N Isobe

**Affiliations:** 1 Kazusa DNA Research Institute, Kisarazu, Chiba, Japan; 2 Institute of Vegetable and Floriculture Sciences, NARO, Tsukuba, Ibaraki, Japan; 3 Forest Tree Breeding Center, Forestry and Forest Products Research Institute, Juo, Hitachi, Ibaraki, Japan; 4 Graduate School of Agricultural and Life Sciences, The University of Tokyo, Bunkyo-ku, Tokyo, Japan; 5 Graduate School of Science, Hiroshima University, Higashi-Hiroshima, Hiroshima, Japan; 6 College of Bioresource Sciences, Nihon University, Kameino, Fujisawa, Kanagawa, Japan

**Keywords:** *Chrysanthemum seticuspe*, genome sequence assembly, linkage map, flowering genes

## Abstract

Cultivated chrysanthemum (*Chrysanthemum morifolium* Ramat.) is one of the most economically important ornamental crops grown worldwide. It has a complex hexaploid genome (2*n* = 6*x* = 54) and large genome size. The diploid *Chrysanthemum seticuspe* is often used as a model of cultivated chrysanthemum, since the two species are closely related. To expand our knowledge of the cultivated chrysanthemum, we here performed *de novo* whole-genome assembly in *C. seticuspe* using the Illumina sequencing platform. XMRS10, a *C. seticuspe* accession developed by five generations of self-crossing from a self-compatible strain, AEV2, was used for genome sequencing. The 2.72 Gb of assembled sequences (CSE_r1.0), consisting of 354,212 scaffolds, covered 89.0% of the 3.06 Gb *C. seticuspe* genome estimated by k-mer analysis. The N50 length of scaffolds was 44,741 bp. For protein-encoding genes, 71,057 annotated genes were deduced (CSE_r1.1_cds). Next, based on the assembled genome sequences, we performed linkage map construction, gene discovery and comparative analyses for *C. seticuspe* and cultivated chrysanthemum. The generated *C. seticuspe* linkage map revealed skewed regions in segregation on the AEV2 genome. In gene discovery analysis, candidate flowering-related genes were newly found in CSE_r1.1_cds. Moreover, single nucleotide polymorphism identification and annotation on the *C*. × *morifolium* genome showed that the *C. seticuspe* genome was applicable to genetic analysis in cultivated chrysanthemums. The genome sequences assembled herein are expected to contribute to future chrysanthemum studies. In addition, our approach demonstrated the usefulness of short-read genome assembly and the importance of choosing an appropriate next genome sequencing technology based on the purpose of the post-genome analysis.

## 1. Introduction

Cultivated chrysanthemum (*Chrysanthemum morifolium* Ramat.), which is produced either as cut flowers or potted and garden plants, is an herbaceous perennial in the family Asteraceae (Compositae). Chrysanthemum was first cultivated in China and developed for horticultural purposes in East Asia. Soon after the discovery of the response of plants to day length, i.e. ‘photoperiodism’,^1^ it was determined that the flowering time of this short-day (SD) plant could be controlled for year-round commercial production by manipulating the day length using blackouts or artificial lighting. Chrysanthemum has also been used in classical physiological studies of photoperiodism, leading to the proposal that floral stimuli (florigens) and floral repressors (antiflorigens) are synthesized in the leaves under inductive/non-inductive photoperiods.^2^^,^^3^ Recent studies have demonstrated that FLOWERING LOCUS T (FT) and its orthologues act as florigens in several species, including chrysanthemum.^4–8^ In 2013, an antiflorigen (CsAFT) was first discovered in a wild chrysanthemum by using a reverse-genetic approach.^9^ An ultra-dense linkage map was recently constructed in cultivated chrysanthemum,^10^^,^^11^ but the complex genome structure of the cultivated chrysanthemum, such as hexaploidy (2*n* = 6*x* = 54), along with the large genome size and self-incompatibility,^12^ have obstructed genetic studies on horticulturally and physiologically important characteristics.

The genus *Chrysanthemum* found in Japan includes 32 species ranging from diploid (2*n* = 2*x* = 18) to decaploid (2*n* = 10*x* = 90).^13^ Diploid *C. seticuspe* (Maxim.) Hand.-Mazz., a wild relative of chrysanthemum, is closely related to cultivated chrysanthemum, with both plants being herbaceous perennial, SD responsive and self-incompatible. Although *C. seticuspe* is generally not thought to be a direct progenitor of the cultivated chrysanthemum, since it is suggested that cultivated chrysanthemum is derived from hybridization between other chrysanthemum species,^14^^,^^15^ it is considered a model species of cultivated chrysanthemum and is thus used for molecular-genetic and physiological analysis.

The recent advances in next genome sequencing (NGS) technology have brought *de novo* whole-genome sequencing to various organisms. The sequencing cost has been dramatically decreasing while the quality of the assembled sequences has been increasing along with the growth of long-read sequencing technologies. However, *de novo* whole-genome sequencing is still costly in species with large genomes, and thus many of these species have not benefitted from the new NGS technologies. In this study, we performed *de novo* whole-genome assembly in *C. seticuspe* by using only the Illumina sequencing platform to achieve low cost assembly. Based on the assembled genome, gene discovery analysis was conducted for genes related to flowering, which is the most important trait in chrysanthemum. Genetic analysis was also performed such as linkage map construction, comparative phylogenetic investigation between *C. seticuspe* and other species, and identification of single nucleotide polymorphisms (SNPs) of cultivated chrysanthemum. Our approach suggests a potential strategy for advancing genetic and genomic studies in species that are not candidates for high quality *de novo* whole-genome assembly due to biological or other difficulties.

## 2. Materials and methods

### 2.1. Plant materials

Three *C. seticuspe* accessions, AEV2, NIFS-0 and NIFS-3, were used in this study. AEV2 is a natural self-compatible mutant that was selfed for five generations to reduce genomic heterogeneity. The resultant line, XMRS10, was used for the whole-genome DNA sequencing. Linkage maps were constructed based on an F_1_ mapping population generated from a cross between NIFS-0 and AEV2. NIFS-3 was used for genome size estimation by flow cytometry and for transcript sequencing for simple sequence repeat (SSR) marker development for linkage map construction.

The genome size of NIFS-3 was estimated in comparison with those of *Arabidopsis thaliana* ‘Columbia’, *Oryza sativa* ‘Nipponbare’, *Dianthus caryophyllus* ‘Francesco’, *Ipomoea nil* ‘Murasaki’, *Eustoma grandiflorum* ‘Piccorosa snow’ and *Zea mays* ‘Honey Bantam Peter 610’ by flow cytometry. Nuclei suspensions of young leaves were prepared using a solution of CyStain PI Absolute P (Partec GmbH, Münster, Germany) added to 2% PVP (polyvinylpyrrolidone)and 15 mM DTT (dithiothreitol) according to the manufacturer’s instructions and analysed with a PAS flow cytometer with 532 nm laser excitation.

### 2.2 Whole-genome sequencing and assembly

Total cellular DNA of XMRS10 was extracted from young leaves by using a DNeasy Plant Mini Kit (Qiagen, Hilden, Germany) and used for construction of paired-end (PE) and mate-pair (MP) libraries. The expected insert size of the PE libraries was 500 bp, and sequences were generated by Illumina HiSeq 2000 and MiSeq (Illumina, San Diego, CA, USA) with read lengths of 101 and 301 nt, respectively (Supplementary Table S1a). Four MP libraries were constructed with expected insert sizes of 2, 5, 10 and 15 Kb and sequenced by HiSeq 2000 with a read length of 101 nt. Sequenced bases with quality scores less than 10 were filtered by PRINSEQ 0.20.4 (see Supplementary Table S2 for information on the bioinformatic tools used in this study), and adaptor sequences in the reads were trimmed using fastx_clipper from the FASTX-Toolkit 0.0.13. The genome size of XMRS10 was estimated by using Jellyfish ver. 1.1.6 based on a k-mer = 17 frequency.

The MiSeq PE reads were assembled by SOAPdenovo2 rev 240 with k-mer = 127, and the gaps were closed by GapCloser 1.10 (*P* = 31, Supplementary Fig. S1). The resultant sequences were subjected to scaffolding with the MP reads using SSPACE2.0 with the parameters of −k 3 and −x 0. The gaps of the scaffolds were closed by GapFiller with the HiSeq PE reads. Potential contaminated sequences on the assembled scaffolds were identified and removed using BLASTN searches against the chloroplast and mitochondrial genome sequence of *A. thaliana* (accession numbers: NC_000932.1 and NC_001284.2), against the human (hg19, https://genome.ucsc.edu/), fungal and bacterial genome sequences registered in NCBI (http://www.ncbi.nlm.nih.gov), against vector sequences from UniVec (http://www.ncbi.nlm.nih.gov/tools/vecscreen/univec/) and against PhiX (NC_001422.1) sequences with E-value cutoffs of 1E-10 and length coverage > 10%. The resultant draft genome sequences were designated CSE_r1.0. Assembly quality was assessed by benchmarking universal single-copy orthologs (BUSCOs) using BUSCO v3.0 software. Known repetitive sequences registered in Repbase and *de novo* repetitive sequences defined by RepeatModeler 1.0.11 were identified by RepeatMasker 4.0.7.

### 2.3. Transcript sequencing, gene prediction and annotation

Total RNAs were extracted from a total of 35 samples of XMRS10 grown under various conditions (Supplementary Table S3). Total RNA was extracted from shoot tips, leaves, young buds, flowers and roots using RNAiso Plus (Takara Bio, Shiga, Japan) followed by an RNeasy Mini Kit (Qiagen) for purification according to the manufacturer’s instructions and LiCl precipitation for the polysaccharide removal if necessary. Libraries were constructed by using a TruSeq standard mRNA HT sample prep kit (Illumina), and sequences were obtained by HiSeq 2000.

Evidence-based gene prediction was first performed by BRAKER1 with the obtained transcript reads and the assembled genome sequences (Supplementary Fig. S2). The predicted genes were used as a training set for ab initio gene prediction by Augustus in the MAKER-P pipeline, along with the additional training sets derived from the following amino acid sequences: *A. thaliana* (TAIR10, https://www.arabidopsis.org/); *D. caryophyllus* (DCA_r1.0)^16^; *Petunia axillaris* (v1.6.2)^17^; *Solanum lycopersicum* (ITAG3.2)^17^; *Populus trichocarpa* (v3.0)^18^; *O. sativa* (IRGSP-1.0)^19^; *Solanum tuberosum* (PGSC DM v3.4)^20^; and *Nelumbo nucifera* (http://lotus-db.wbgcas.cn, downloaded June 2016). In parallel, evidence-based gene prediction was performed by mapping the *C. seticuspe* transcript reads onto the assembled genome sequences using the Tophat-Cufflinks pipeline and merged with the results of ab initio gene prediction by the MAKER-P pipeline. The resultant sequences were filtered by sequence length and annotation edit distance (AED) score, and then subjected to homology searches against the NCBI NR database (http://www.ncbi.nlm.nih.gov) and *A. thaliana* in TAIR11 using BLASTP with an E-value cut-off of 1E-20. Domain search was conducted by InterProScan against the InterPro database with an E-value cutoff of 1.0. Transposable elements (TEs) were identified by BLAST searches against the NCBI NR database and domain searches against InterPro and GyDB 2.0 with an E-value cutoff of 1.0. The sets of sequences showed significant similarity against the databases, with the exception that TEs were designated as CSE_r1.1_cds. Functional analysis of coding DNA sequences (CDSs) in CSE_r1.1_cds was performed by classifying the sequences into the plant gene ontology (GO) slim categories and the ‘euKaryotic clusters of Orthologous Groups’ (KOG) categories, and mapping onto the Kyoto Encyclopaedia of Genes and Genomes (KEGGs) reference pathways. Transfer RNA genes were predicted using tRNAscan-SE ver. 1.23 with default parameters. rRNA genes were predicted by BLAST searches with an E-value cutoff of 1E-10. The *A. thaliana* 5.8S and 25S rRNAs (accession number: X52320.1) and 18S rRNA (accession number: X16077.1) were used as query sequences.

### 2.4. Gene discovery analysis related to flowering

The genes related to flowering of *C. seticuspe* were identified based on the BLAST searches and OrthoMCL analysis (see below). The amino acid sequences of flowering pathway genes of Arabidopsis were used as query for tblastn search against CSE_r1.1_cds and CSE_r1.1_maker_cds. The phylogenetic tree of the phosphatidylethanolamine-binding proteins (PEBPs) was constructed using the Neighbour-Joining method in MEGA7 software based on the amino acid sequences of PEBP from the plant species, Al: *Arabidopsis lyrate*, Am: *Antirrhinum majus*, At: *A. thaliana*, Bv: *Beta vulgaris*, Ci: *Citrus unshiu*, Cs: *Chrysanthemum seticuspe*; Ej: *Eriobotrya japonica,* Fv: *Fragaria vesca,* Gm: *Glycine max,* In: *Ipomoea nil,* Le: *Lycopersicon esculentum,* Lp: *Lolium perenne,* Md: *Malus* × *domestica,* Nt: *Nicotiana tabacum,* Os: *O. sativa,* Pc: *Pyrus communis,* Pn: *Populus nigra,* Pp: *Physcomitrella patens,* Ps: *Pisum sativum*, Ro: *Rosa chinensis,* Sm: *Selaginella moellendorfii,* Ta: *Triticum aestivum* and Vv: *Vitis vinifera.* Bootstrap values for 1,000 resampling are shown on each branch.

Of the investigated candidate genes, three genes—*CEN-like* (Cse_sc005398.1_g060.1), *CsAFL1* and *CsFL*—were subjected to gene expression analysis in rosette-forming *C*. *seticuspe* line NIFS-3. The rosette-forming plants were divided into two groups, a non-chilled group and a chilled (5°C, 4 weeks) group. After growing the plants under the respective conditions (Supplementary Fig. S3), shoot tips from each group were sampled for RNA extraction. Total RNA were extracted with an RNA extraction plant mini kit (Qiagen). The expression levels of the three genes were investigated using quantitative real-time PCR. The primers used were as follows. For the *CEN*-like gene: qRT-f, AGGATTCATCAATCCATGTG; and qRT-r, CCTCCGTGAATATCAACCCG; for *CsAFL1* (the *AP1/FUL*-like gene): *CsAFL1*-QF, TGATCCCTACATCAGTGCTCCA; and *CsAFL1*-QR: CAAGGGGGCAAGACTATTGATG; and for *CsFL* (a homologue of *LEAFY*): *CsFL*-QF, CTTTGTCATGCTGAACGGAGTG; and *CsFL*-QR, GAGCATATGACCAACACCACCA. *CsACTIN* (AB770470) was used as an internal standard to normalize raw data.

### 2.5. Linkage-map construction

Linkage maps were constructed based on a F_1_ mapping population generated as a cross between NIFS-0 and AEV2. SNP genotyping by ddRAD-Seq analysis was performed for 84F_1_ individuals and the parental lines as described by Shirasawa et al.^21^ The ddRAD-Seq libraries were constructed with a combination of restriction enzymes, *Pst*I and *Msp*I and sequenced by MiSeq with a read length of 251 nt. The reads were mapped onto CSE_r1.0 by Bowtie2, and variant calls were performed by SAMtools 0.1.19 and VarScan 2.3 as described by Shirasawa et al.^21^ The candidate SNPs were filtered under the following conditions: exclude in/dels, minimum depth = 5, minimum quality = 10, max missing = 0.5, minor allele frequency = 0.2, polymorphic between the parental lines, and exclude AEV2 = Alt (alternative) homozygous.

Segregation analysis was also performed using the SSR markers developed in this study. A total of 4,056,757 transcript sequences of leaves and shoot apices of NIFS-3 were obtained by Roche 454 FLX (Roche Diagnostics, Indianapolis, IN, USA) and assembled by Newbler 2.6 (Roche Diagnostics). SSR motifs on the assembled sequences were identified using SciRoKo software and the Fuzznuc tool from EMBOSS. Primers were designed by Primer3 for the SSRs that had 8, 5, 4, 3 and 3 repeats in the di-, tri-, tetra-, penta- and hexa-motifs, respectively. Polymorphic analysis of the SSR markers was performed using an ABI 3730xl fluorescent fragment analyser (Applied Biosystems, Foster City, CA, USA) or 10% polyacrylamide gel electrophoresis according to Isobe et al.^22^

For linkage analysis, the segregating SNPs were grouped using MSTmap with the following parameters: cut-off *P*-value = 1, no map size = 0; the SSR loci were separately grouped by JoinMap 4. Then, correspondent SNP and SSR linkage groups (LGs) were identified and integrated based on segregation patterns in the mapping population. The segregation data was categorized into two parent-specific datasets and ordered by JoinMap 4 under the following conditions: Kosambi’s mapping function, LOD (logarithm of the odds) >1.0, REC frequency < 0.4, goodness of fit jump threshold for removal of loci = 5.0, number of added loci after which a ripple is performed = 1, and third round = yes.

### 2.6. Comparative and phylogenetic analysis

The translated protein sequences of CSE_r1.1_cds were clustered by OrthoMCL with those of *A. thaliana* (Araport11, https://www.araport.org/data/araport11/), *S. lycopersicum* (tomato, ITAG3.2),^17^*Lactuca sativa* (lettuce, Lsativa_467_v5, http://lgr.genomecenter.ucdavis.edu/)^23^ and *Helianthus annuus* (sunflower, Ha412v1r1_port_v1.0)^24^ with the parameters c = 0.6 and aL = 0.9. The phylogenetic analysis was performed by MEGA 7.0.9 beta and TIMETREE based on the single copy genes conserved in *C. seticusp*e and the four species. The divergence time of 104 MYA between *A. thaliana* and *S. lycopersicum* was used for calibration. The published sequence read archive (SRA) transcripts for six cultivated chrysanthemum (*C. × morifolium*) varieties were mapped onto CSE_r1.0 by TopHat v2.1.1 (Supplementary Table S1b) in order to investigate the mapped ratios and identify variants. Variants were called based on the mapping result using SAMtools 0.1.19 and subsequently filtered using VarScan 2.3. SNP effects on gene function were predicted by using SnpEff ver.4.0.

## 3. Results and discussion

### 3.1. Whole-genome sequencing and assembly of *C. seticuspe*

A total of 94.2 Gb of MiSeq PE and 137.5 Gb of HiSeq PE reads were subjected to k-mer frequency analysis (Supplementary Fig. S4). Two peaks were observed in the distribution of the number of distinct k-mers at a given multiplicity distribution. Based on the multiplicity value of the higher peak (multiplicity = 67), the genome size of XMRS10 was estimated as 3.06 Gb. This estimated genome size was close to 2.90 ± 0.03 Gb, which was the estimated genome size of NIFS-3 by flow-cytometry analysis. The large difference in height between the two peaks (multiplicity = 67 and 134) suggests that most of the XMRS10 genome is homozygous; however, heterozygous regions still remain after selfing five times from AEV2.

The 94.2 Gb MiSeq PE reads were assembled by SOAPdenovo2 (k-mer = 127) and GapCloser, and 6,480,313 scaffolds were generated with a total length of 3,189, 146,773 bp (Supplementary Table S4). Then, the sequences were further scaffolded by SSPACE2.0 with MP reads, and gaps were filled with HiSeq PE reads by GapFiller. After excluding contaminated sequences and those shorter than 500 bp, the remaining 354,212 sequences were designated as a draft genome sequence, CSE_r1.0, and subjected to analysis (Table 1). The total and N50 lengths of CSE_r1.0 were 2,721, 839,164 and 44,741 bp, respectively. The assembled sequences covered 89.0% of the XMRS10 genome when the genome size was estimated as 3.06 Gb. The CSE_r1.0 sequences were mapped onto the 1, 440 BUSCOs to assess the sequence quality. The number of complete BUSCOs was 1, 279 (88.8%), including 1,153 (80.1%) single copy genes and 125 (8.7%) duplicated genes. The numbers of fragmented and missing BUSCOs were 40 and 121, respectively (Supplementary Table S5). Although the assembled genome sequences were fragmented, the BUSCO results suggested that the CSE_r1.0 had an acceptable quality for gene prediction and annotation.

**Table 1:** Statistics of the *C. seticuspe* genome assembly and CDS

	CSE_r1.0	CSE_r1.1_maker_cds	CSE_r1.1_cds
	Genome	CDS	CDS (after filtering)
Number of sequences	354,212	158,375	71,057
Total length (bp)	2,721,839,164	188,275,935	89,043,396
Average length (bp)	7,684	1,189	1,253
Maximum length (bp)	447,582	16,482	16,482
Minimum length (bp)	500	300	300
N50 length (bp)	44,741	1,560	1,584
GC%	36.0	41.1	40.8
Repeat %	72.5	—	—
Number of complete genes	—	15,796	15,304
Number of partial genes	—	142,579	55,753
TEs	—	55,741	—

The total length of repetitive sequences in CSE_r1.0 was 1, 774 Mb, and these sequences occupied 72.5% of the *C. seticuspe* genome (CSE_r1.0, Supplementary Table S6). The ratio of repeat sequences was similar to that for lettuce (77.5%), which has an estimated genome size of 2.5 Gb.^23^ The ratio of unknown repeat sequences in CSE_r1.0 was higher than those in the sunflower and lettuce genomes. The total length of known repeats was 928 Mb, and Class I (long terminal repeat) elements were most frequently observed.

### 3.2. Gene prediction and functional analysis

A total of 717,973 genes were identified by the MAKER-P pipeline. Because the number of predicted genes was large, it was considered that a large number of suspicious gene sequences were included in the results. The AED score was therefore calculated for each gene, and filtering of the gene sequences was performed by AED score (<0.5) and sequence length (≥300 bp). The resultant number of predicted genes was 158,374 and designated as CSE_r1.0_maker_cds. The gene sequences with more accurate estimations were further selected by means of a BLASTP search against the NCBI NR database and InterProScan. The remaining 71,052 putative genes were designated as CSE_r1.0_cds. The number of identified tRNA- and rRNA-encoding genes was 2,738 and 100, respectively (Supplementary Table S7).

In order to investigate the quality of the predicted genes, a BLASTN search (E-value cut-off of 1E-10 and length coverage > 10%) was performed for 47 previously identified genes involved in flowering-time regulation in *C. seticuspe* NIFS-3.^8^^,^^9^^,^^25^^,^^26^ A total of 44 genes were found in CSE_r1.0_maker_cds, and three were missing (Supplementary Table S8). Of the 44 identified genes, four were tagged as TE, and not included in CSE_r1.0_cds. Thirteen sequences predicted by BRAKER1 showed higher similarity than those in CSE_r1.0_maker_cds. CsFTL2, one of the three missing genes, was found adjacent to CsFTL1 in one scaffold. PCR and Sanger sequencing showed that the physical distance between these genes was 2.5 kbp (Supplementary Fig. S5). The CDS identity of these genes was 96.4%.^8^ By using GeneScan, CsFTL2 was successfully predicted. The sequences or tags were replaced or added, and the revised sets of gene sequences were designated CSE_r1.1_maker_cds and CSE_r1.1_cds (Table 1).

The results of gene prediction often differ according to the methods used, and there is no single best solution. Therefore, we combined multiple methods for gene prediction by MAKER-P and filtered the suspicious sequences. Although some genes were miss-classified as TE and the results of BRAKER1 were sometimes better than those of MAKER-P, the number of missing genes was only three of 47. In addition, there was a possibility that the two genes that were not identified by GeneScan do not exist in the XMRS10 genome. Hence, we concluded that CSE_r1.1_maker_cds covered most of the genes in XMRS10, but some of the genes might have been missed in CSE_r1.1_cds.

The numbers of complete and partial genes in CSE_r1.1_cds were 15,304 and 55,753, respectively, while those in CSE_r1.1_maker_cds were 15,796 and 142,579 (Table 1). The putative genes (CDSs) having similarity to TE made up 35.2% of CSE_r1.1_maker_cds (Supplementary Table S9). The CDSs were tagged with ‘f’, ‘p’ and ‘d’ according to their level of similarity against the NR database (f: E-values ≤ 1E-20 and identity ≥ 70%; p: E-values ≤ E-20 and identity <70%) and InterPro (d: *E*-values ≤ 1.0). Of the 71,057 CSE_r1.1_cds, 55,197 (77.7%) CDSs were tagged ‘f’ (15,304 CDSs) or ‘p’ (39,893 CDSs). The number of CDSs tagged ‘d’ was 65,317.

Functional analysis was performed for CSE_r1.1_cds by classifying the putative genes into the GO, KOG and KEGG databases. A total of 33,372 (47.0%) genes were annotated with GO categories, including 19,116 genes involved in biological processes, 6,780 genes coding for cellular components and 29,988 genes associated with molecular function (Supplementary Fig. S6). The ratio of annotated genes was less than in the other four compared species, i.e. sunflower, lettuce, tomato and *A. thaliana.* The genes in these four species were predicted based on pseudomolecules, while the *C. seticuspe* genes were predicted based on 354,212 scaffolds. Hence, it was considered that the *C. seticuspe* genes predicted in this study were more fragmented than the genes of the four other species used for comparison, and the fragmentation caused the lower ratio of annotated genes. A total of 54,735 genes showed significant similarity to genes in the KOG database, and the distributions of the KOG functional categories were similar to those of the compared species (Supplementary Fig. S7). The total number of mapped genes on the KEGG pathway was 4,941 (Supplementary Table S10).

### 3.3. Gene discovery analysis related to flowering

Photoperiodic flowering is an important characteristic controlling the flowering time in the commercial production of chrysanthemums. In contrast to the model plant species such as *Arabidopsis* and rice, photoperiodic flowering of chrysanthemums is dependent on the absolute duration of darkness and is sensitive to light exposure at midnight.^9^ The majority of the photoperiod-dependent flowering pathway genes found in Arabidopsis, such as photoreceptors, circadian clock components or clock-controlled transcription factor genes, have been identified in the *C. seticuspe* genome (Supplementary Table S11). This indicates that the basic components in the photoperiodic flowering pathway are well conserved even among species with different photoperiodic responses, but that the regulation of each gene might be different or that a unique, alternative pathway might also exist in chrysanthemums.

**Figure 1:**
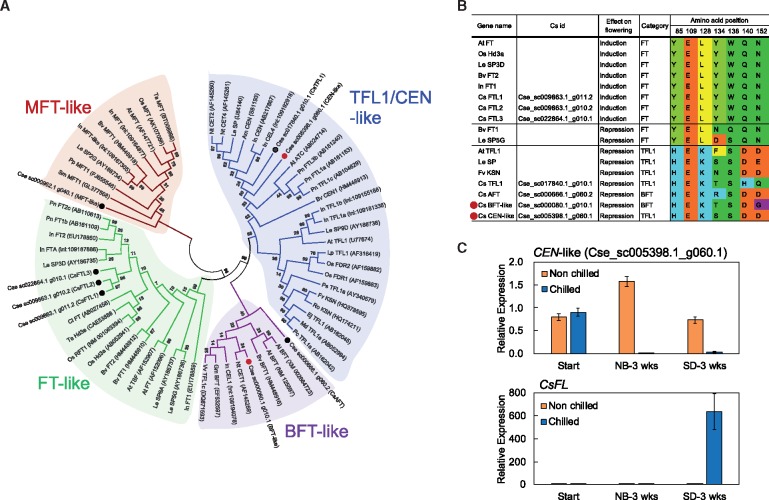
Gene discovery analysis related to flowering. (A) Phylogenetic tree of PEBP family proteins from the plant species including *C. seticuspe* homologues. The tree was constructed using the Neighbor-Joining method in MEGA7 software. Bootstrap values for 1,000 re-samplings are shown on each branch. Black and red dots represent *C. seticuspe* homologues identified in previously and the present studies, respectively. (B) Amino acid alignment of FT/TFL1 family proteins at the conserved positions that determine inducer or repressor activities. (C) Effects of chilling on expression of the newly identified *CEN*-like gene (Cse_sc005398.1_g060.1) and a floral meristem identity gene, *CsFL* (a homologue of LEAFY) in the rosette-forming plants. Data are means ± SE (*n* = 5). The value of a non-chilled plant at the start of the experiment was set to one.

Photoperiodic floral initiation of chrysanthemums is regulated by the antagonistic action of two homologous FT/TERMINAL FLOWER 1 (TFL1)-like proteins. Three *FT*-like genes (*CsFTL1*, *CsFTL2* and *CsFTL3*) and two *TFL1/BFT*-like genes (*CsAFT* and *CsTFL1*) were identified in *C. seticuspe* in previous reports.^8^^,^^9,^^27^ Two additional *TFL1*-like genes and one *MFT*-like gene were newly identified in this study, but no additional *FT*-like genes were found in the *C. seticuspe* genome (Fig. 1A). Phylogenetic analysis and amino acid alignment using the PEBPs from other plant species suggested that only three *FT*-like genes (*CsFTL1*, *2* and *3*) have floral promoter activity, while four *TFL1/CEN/BFT*-like genes have floral repressor activity (Fig. 1B).^28^

Chrysanthemum plants form rosettes by cessation of shoot elongation in autumn. This habit of chrysanthemum is considered to be a type of dormancy adaptation in the annual cycle between growth and dormancy of herbaceous perennials.^29^ Flowering is blocked in dormant rosette-forming chrysanthemums even under floral inductive day-length conditions. This flowering incompetence is reversed by chilling, somewhat as in a vernalization response.^29^ It is noteworthy that the newly identified *CEN*-like gene (Cse_sc005398.1_g060.1) is highly expressed in the shoot tips of rosette-forming dormant plants that are incompetent to initiate flowering (Fig. 1C). In the chilled plants where expression of the *CEN*-like gene was suppressed to a low level, the expression of two floral meristem identity genes (*CsAFL1* and *CsFL*) of *C. seticuspe* were up-regulated within three weeks after the transfer from the non-inductive NB to inductive SD conditions (Fig. 1C and Supplementary Fig. S3). The expression pattern of the *CEN*-like gene indicates that this gene might be involved in the seasonal change of flowering competence in chrysanthemum.

### 3.4. Linkage map construction

In order to demonstrate an application of the assembled *C. seticuspe* genome to genetic analysis, *C. seticuspe* linkage maps were constructed based on an F_1_ mapping population derived from a cross between AEV2 and NIFS-0 based on ddRAD-Seq and SSR markers. The total bases of the ddRAD-Seq reads in each F_1_ individual were 43.8 Mb (203, 321 reads) on average. The reads were mapped onto CSE_r1.0 with a mean mapped ratio of 83.4%, and a total of 5,206 SNPs were identified. Meanwhile, a total of 66,438 assembled sequences were generated by Newbler2.6 from 2,626,286 454/FLX transcript sequences. Based on the 66,438 sequences, 9,605 primer pairs were designed on the flanking regions of SSRs identified by Fuzznuc (Supplementary Table S12). The existence of SSRs between the primer sequences was further investigated by SciRoKo, and the confirmed 3,140 SSR markers were subjected to the subsequent polymorphic analysis.

**Figure 2:**
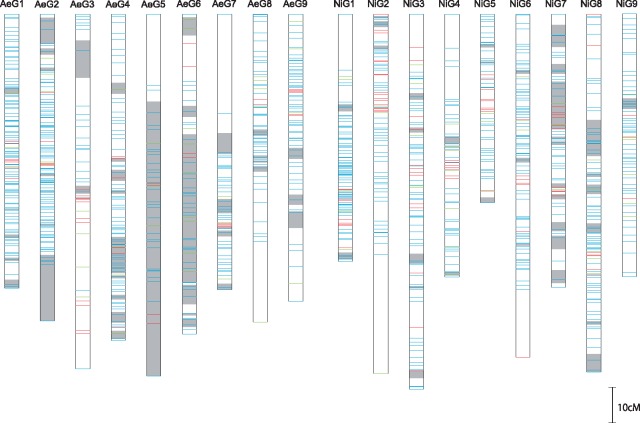
AEV2 (left) and NIFS-0 (right) bin linkage maps. Blue, red and green bars show locus positions mapped by SNPs, SSR markers and both types of markers. Grey bars represent regions where mapped loci showed significant segregation distortion (*P* ≤ 0.05).

Parent-specific linkage maps were generated for AEV2 and NIFS-0 with nine LGs in each. The number of mapped loci was 2,124 in the AEV2 map, while that in the NIFS-0 map was 2,239 (Supplementary Tables S13 and S14). The number of bins (non-identical locus positions) in AEV2 and NIFS-0 was 581 and 615, respectively. A higher ratio of segregation distortion was observed in the AeG5 and AeG6 LGs in the AEV2 map (Fig. 2). The reference alleles were more frequently observed at the distorted loci, particularly on AeG5, suggesting the existence of a biological bias in meiosis or chromosome pairing in AEV2.

### 3.5. Comparison with other plant species at the gene level

**Figure 3:**
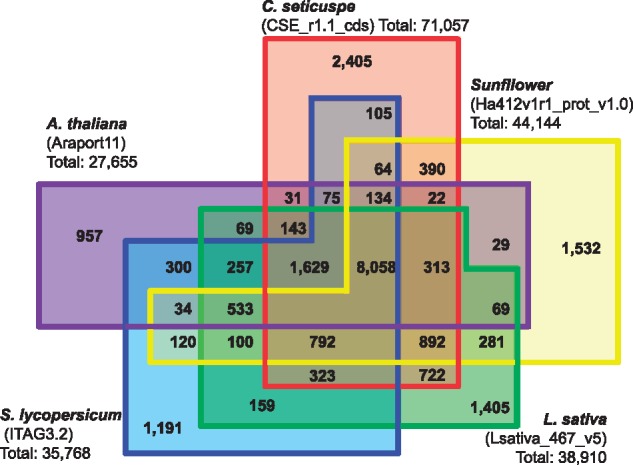
Venn diagram showing numbers of gene clusters in *C. seticuspe*, sunflower (*H. annuus*), lettuce (*L. sativa*), tomato (*S. lycopersicum*) and *A. thaliana*.

The translated protein sequences in CSE_r1.1_cds were clustered and compared with the protein sequences in other plant species (sunflower, lettuce, tomato and *A. thaliana*) at the amino acid level by OrthoMCL. Of the 71,057 genes in CSE_r1.1_cds, 59,927 genes were classified into 16,098 clusters and 2,405 were classified into *C. seticuspe*-specific clusters. A total of 19,979 (33.3%) of the genes were clustered with all four of the plant species compared (Fig. 3, Supplementary Fig. S8). The finding that a larger percentage of *C. seticuspe* genes was clustered exclusively with sunflower genes (10.0%) compared to the percentage clustered exclusively with lettuce genes (3.5%) suggested that, between these two species in the family *Asteraceae*, sunflower showed the greater similarity to *C. seticuspe*. The family Asteraceae has been classified into 12 subfamilies,^30^ and the genera Chrysanthemum and Helianthus belong to the subfamily *Asyeroideae*, while the genus *Lactuca* belongs to the subfamily Cichorioideae. These results agree with the previous taxonomy studies.

**Figure 4:**
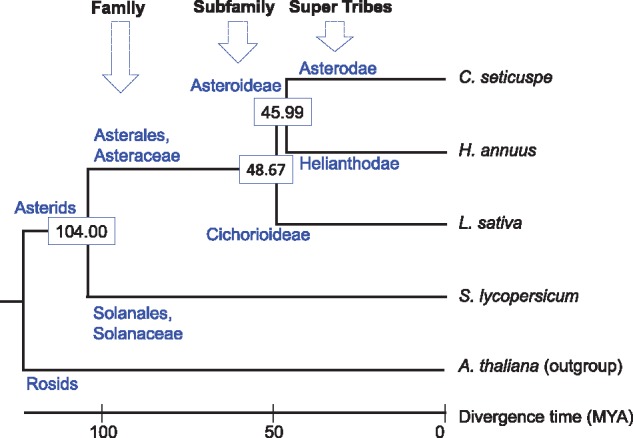
A phylogenetic tree based on the 2,280 common single-copy genes of the four *Asterids* species and *A. thaliana*.

Phylogenetic analysis was further performed with the 2,280 single copy genes observed in the four Asterids species (*C. seticuspe, H. annuus, L. sativa* and *S. lycopersicum*) and *A. thaliana,* as *A. thaliana* was used for the outgroup (Fig. 4). Our results showed that the subfamilies Asterodeae and Cichorioideae diverged ∼48.67 MYA, and this estimate agreed with the previous study.^24^ The divergence time between super tribes Asterodeae and Helianthodae was estimated as ∼45.99 MYA, 2.68 MYA after the divergence of the subfamilies Asterodeae and Cichorioideae.

### 3.6. SNP mining and identification of variants in hexaploid-cultivated chrysanthemums

Despite their importance as ornamental plants, cultivated chrysanthemums (*C. × morifolium*) are rather difficult to study at a genetic level due to their autohexaploidy, large genome size and self-incompatibility. Diploid genome sequences are often used as references of genetic and genomic analysis in polyploid species, such as rose (*Rosa hybrida*), strawberry (*Fragaria* × *ananassa*) and sweetpotato (*Ipomoea batatas*).^31–33^ The genome sequence of *C. seticuspe*, a diploid wild relative, can shed light on the complex nature of the cultivated chrysanthemum genome as a reference plant for chrysanthemum species.

The transcript sequences of the six cultivated chrysanthemums (Supplementary Table S1b) were mapped onto the genome sequences of *C. seticuspe* and variants were identified in order to investigate the usefulness of the *C. seticuspe* genome for genetic studies in cultivated chrysanthemums. A total of 20.4–156.9 M reads of each variety were mapped onto CSE_r1.0. The mapped ratio of the reads ranged from 56.7% (Jinba) to 82.5% (Nannongxunzhang) (Supplementary Table S15a). Two factors were considered as the causes of the large difference in the mapped ratios: read length differences in the six cultivars (49∼100 nt; Supplementary Table S1b) and different sequence similarities against *C. seticuspe*. The total number of SNPs identified among the six varieties was 954,706, and 294,601 SNPs were identified in all six varieties (Supplementary Table S15b). The remaining 660,105 SNPs (69%) were missing in one to five of the varieties, implying the existence of large gene sequence variation among cultivated chrysanthemums.

The SNP genotype call was performed for the 294,601 SNPs that were identified in all six varieties with consideration for the percentages of reads having reference (Ref) and alternative (Alt) bases. SNPs with Alt read ratios of 8.35–91.65 were designated as hetero SNPs. A total of 27, 855 SNPs exhibited a uniform genotype among the six varieties (Supplementary Table S15c). These SNPs were considered to represent the variants between *C. seticuspe,* XMRS10, and cultivated chrysanthemum. The functions of the SNPs were annotated by SnpEff based on their positions in the mapped gene sequences (Supplementary Table S16 and Fig. S9). Of the total 22,470 genes, 14,741 were identified as SNPs with multiple impacts and 533 were identified as high-impact SNPs. As a result of breeding, cultivated chrysanthemums possess a wider variety of characteristics than *C. seticuspe*—including with respect to flowering time, flower shape and colour. Causative variants for the characteristics of cultivated chrysanthemums might be identified by further analysis of the functions of the SNPs.

The results described above showed that the *C. seticuspe* genome is applicable to genetic and gene functional analyses in cultivated autohexaploid chrysanthemums. In autohexaploid sweetpotatoes, single-dose SNP markers were detected by using the genome sequence of diploid *I. trifida* and selected according to the allelic dose for each SNP locus, and a high density genetic map consisting of single-dose SNP markers was constructed.^33^ The technique is also applicable to high-density mapping for genetic improvement in cultivated chrysanthemums. A large number of genome-wide SNP markers are expected to be produced by mapping RAD-seq or other sequence data of cultivated chrysanthemum onto the assembled genome sequences, CSE_r1.0.

## 4. Conclusions

In this study, *de novo* whole-genome assembly was performed in the genus *Chrysanthemum* with Illumina reads only. Various NGS technologies have recently become available at reasonable cost, although some of these technologies are still expensive for organisms with large genomes. Illumina reads are the lowest-cost NGS technology, and unlike long-read technologies, Illumina does not require large computer resources for assembly. Although in this study the numbers of assembled sequences and predicted genes indicated that the sequences were still fragmented, the generated draft genome and linkage maps revealed the features of the *C. seticuspe* genome. In addition, a candidate flowering-related gene, the CEN-like gene (Cse_sc005398.1_g060.1), was newly discovered based on the assembled genome. Moreover, our results demonstrated the applicability of the *C. seticuspe* genome for genetic and gene functional analysis in cultivated autohexaploid chrysanthemums (*C.* × *morifolium*). The genome and gene sequences generated in this study are thus expected to contribute to future chrysanthemum investigations. In addition, our approach demonstrated the usefulness of short reads genome assembly and the importance of choice of NGS technologies depending on the purpose of post-genome analysis.

## 5. Data availability

The genome assembly data, annotations, gene models and SSRs on the linkage map are available at the MumGARDEN (http://mum-garden.kazusa.or.jp/). The obtained genome sequence reads are available from the DDBJ Sequence Read Archive (DRA) under the accession number DRA005542. The BioProject accession number of the study is PRJDB5536.

## Supplementary Material

dsy048_Supplementary_Data
