# Supplementary material for: De novo whole-genome assembly in Chrysanthemum seticuspe, a model species of Chrysanthemums, and its application to genetic and gene discovery analysis
Source: DNA Res. 2019 Jan 27;26(3):195–203. doi: 10.1093/dnares/dsy048 (PMC6589549; doi:10.1093/dnares/dsy048)
Supplement: dsy048_Supplementary_Data [file dsy048_supplementary_data.zip › dsy048-Suppl_data/Supplementary Figures.pdf]

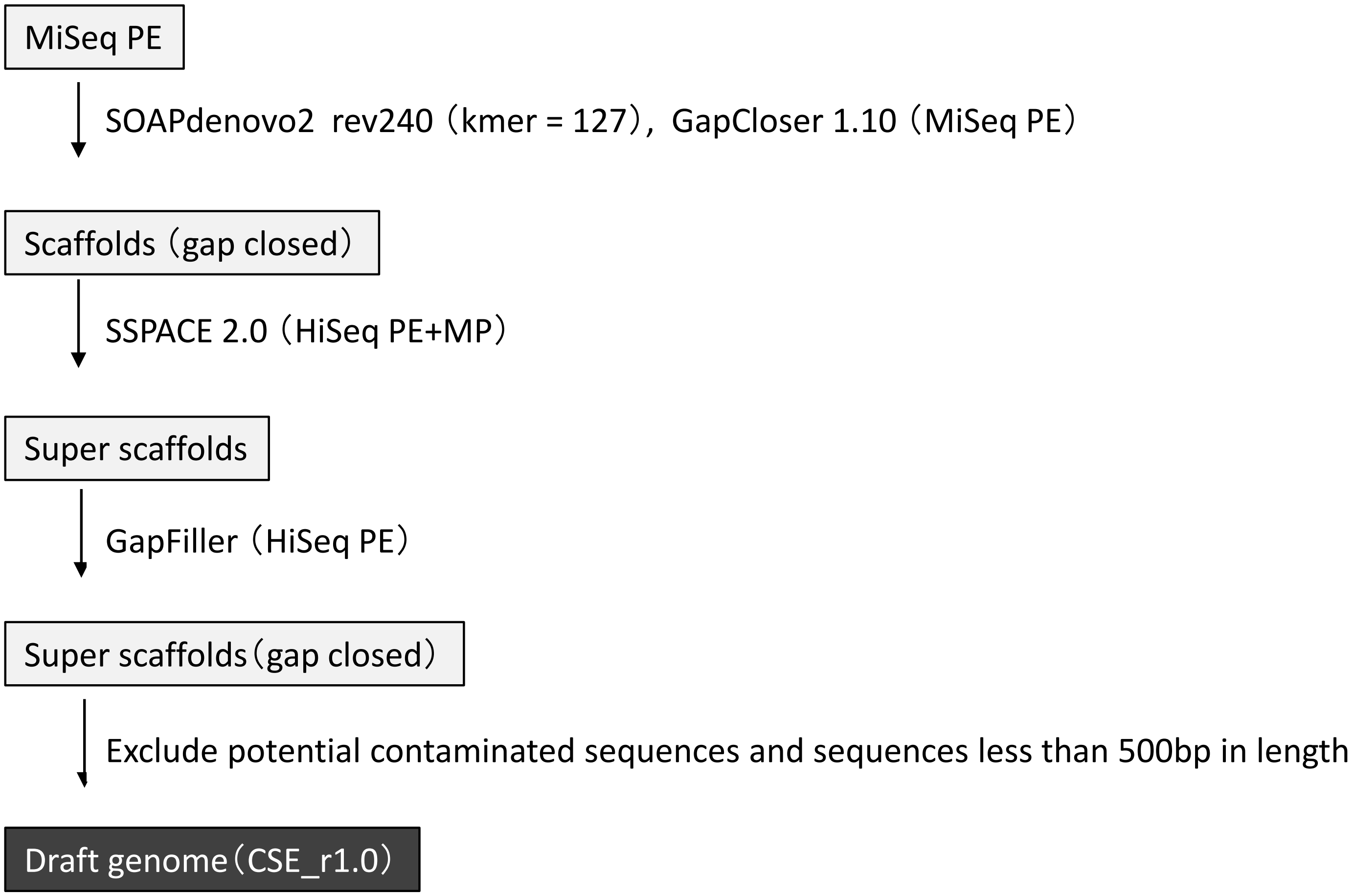

Supplementary Fig. S1. The strategy of whole genome assembly of CSE_r1.0. PE and MP represent paired-end and mate-pair reads, respectively.

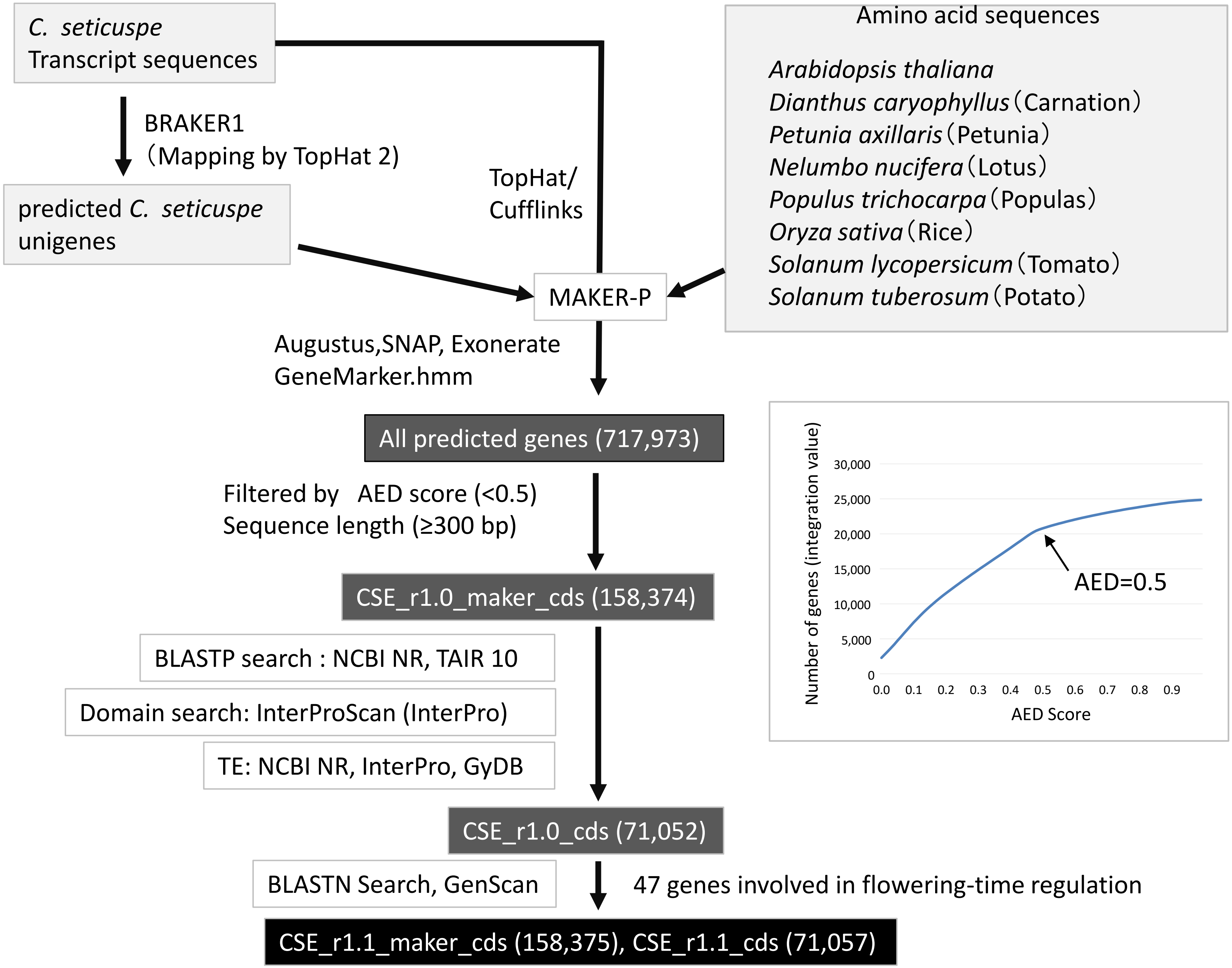

Supplementary Fig. S2. The strategy of gene prediction of CSE_r1.0.

A)

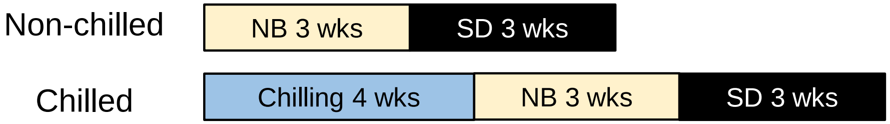

B)

	Flowering (%)	Days to flower bud visible
Non chilled	8	36.5 ± 2.45
Chilled	100	26.8 ± 0.44

C)

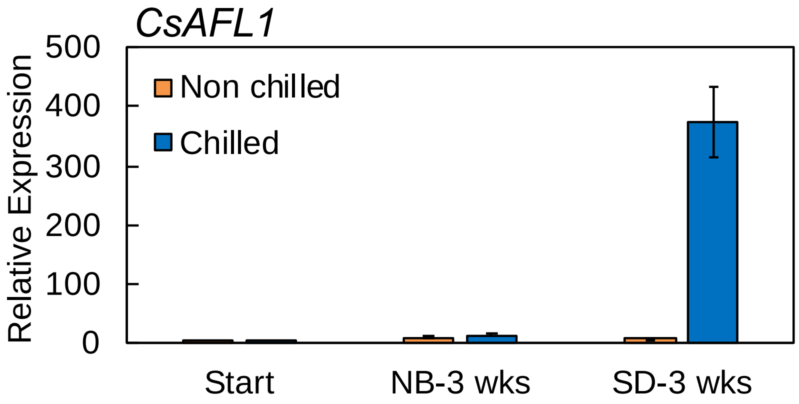

Supplementary Fig. S3
Effects of chilling on flowering and on expression of *CsAFL1, a* floral meristem identity gene in the rosette-forming plants. **A)** The plants were grown at 20°C/15°C with an 8h short day (SD) with 2h night break (NB) conditions for 3 weeks, then transferred into a 8h SD conditions. The chilled plants were subjected to chilling (5°C, 4 weeks) before NB. **B)** Effects of chilling on flowering in the rosette-forming plants. **C)** Comparisons of gene expression of *CsAFL1,* . Data are means ± SE (n = 5). The value of a non-chilled plant at the start of the experiment was set to one.

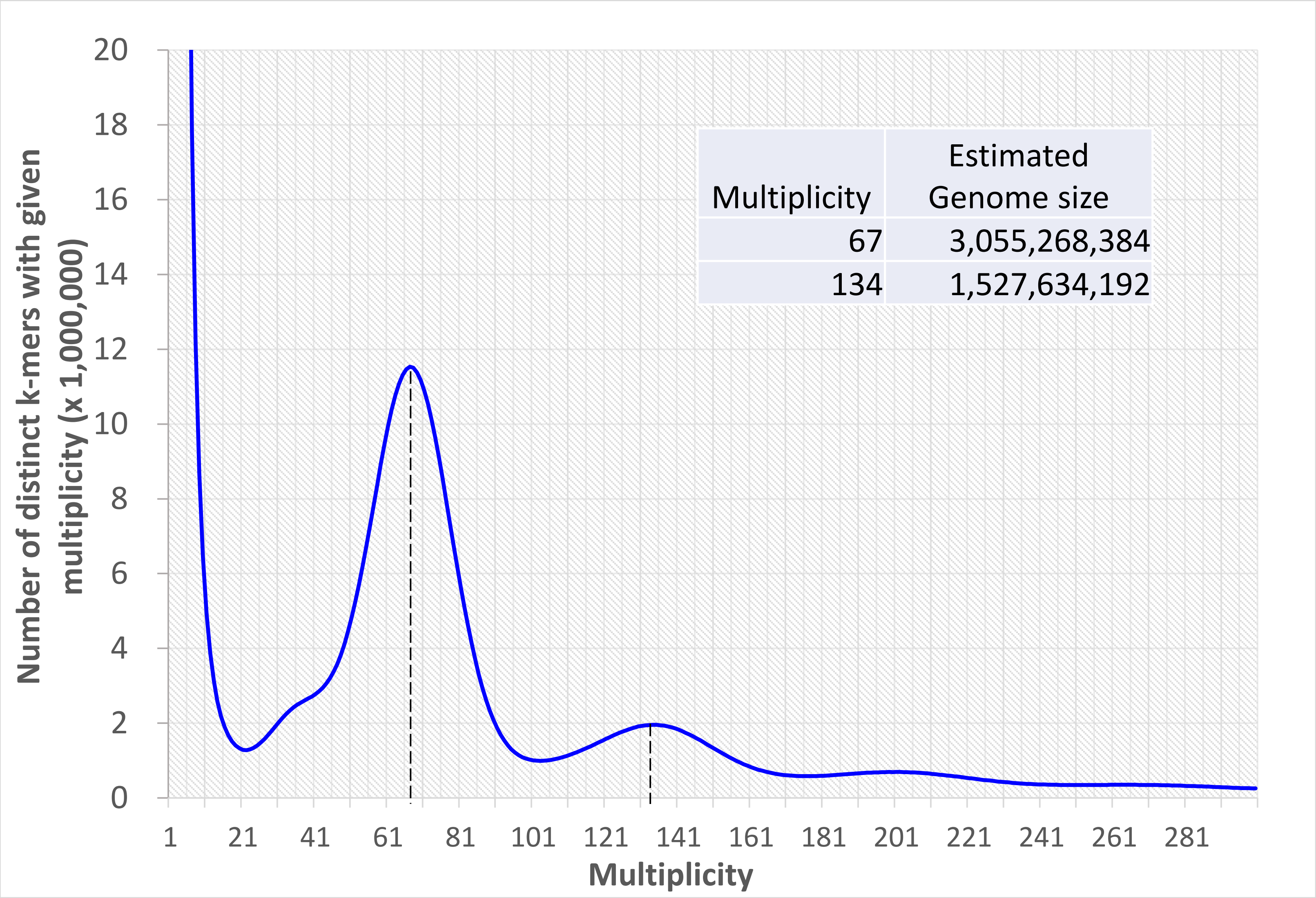

Supplementary Fig. S4. Genome size estimation with the distribution of the number of distinct kmers (kmer = 17) with the given multiplicity values.

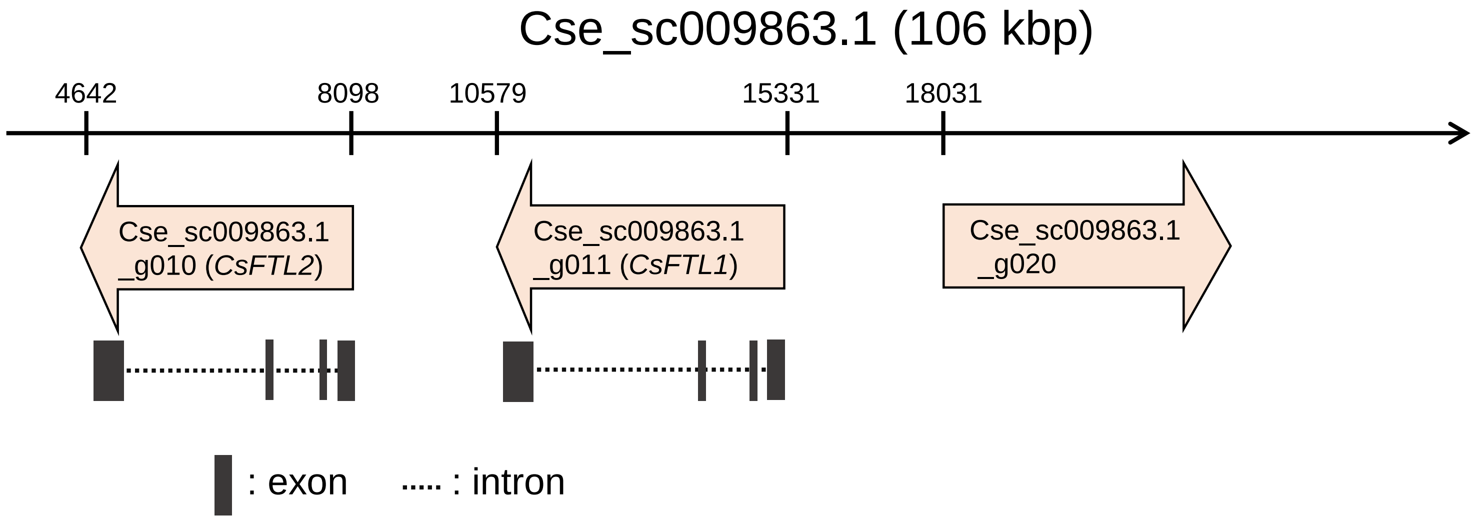

Supplementary Fig. S5. Structures of tandemly arrayed *FTL* genes in *C. seticuspe*.

A: Numbers and ratios of gene hits against the GO database

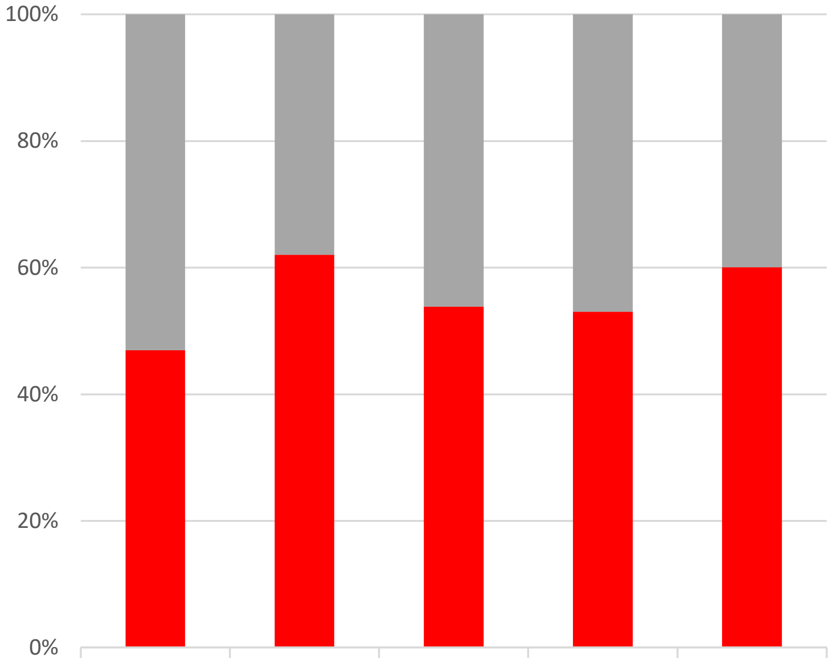

	C. seticuspe	*H. annuus*	*L. sativa*	*S. lycopersicum*	*A. thaliana*
Not annotated	*37,685*	*16,759*	*17,959*	*16,807*	*11,056*
Annotated	*33,372*	*27,385*	*20,951*	*18,961*	*16,609*

Annotated   Not annotated

B: Ratio of the classified GO functional categories (Root)

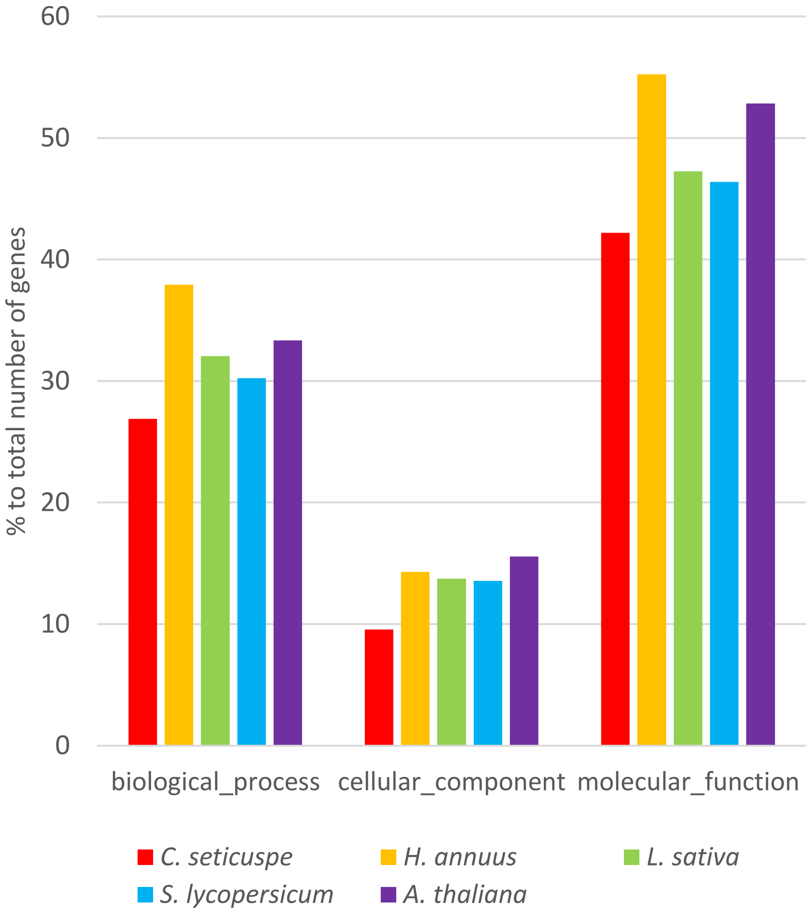

Supplementary Fig. S6. A: Numbers and ratios of genes annotated by the GO database in *C. seticuspe* (CSE_r1.1_cds), sunflower (*H. annuus,* Ha412v1r1), lettuce (*L. sativa,* v5), tomato (*S. lycopersicum,* ITAG3.2) and *A. thaliana* (Araport11). **B:** Ratio of the classified GO categories in the predicted genes.

A: Numbers and ratios of gene hits against the KOG database

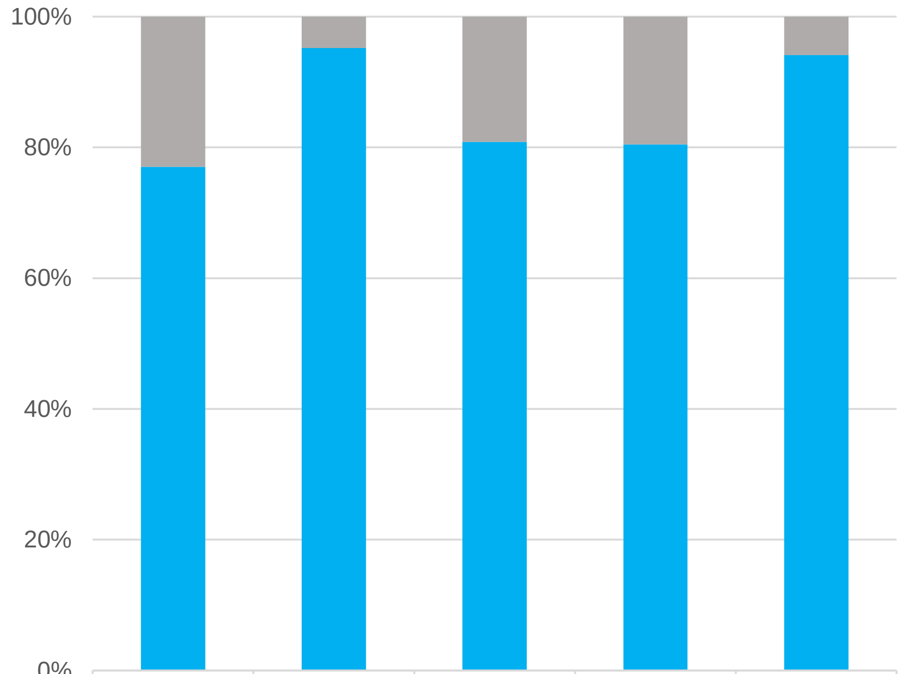

	C. seticuspe	*H. annuus*	*L. sativa*	*S. lycopersicum*	*A. thaliana*
No hits	16,322	2,128	7,448	6,992	1,629
Hits	54,735	42,016	31,462	28,776	26,036

B: Ratio of the classified KOG functional categories

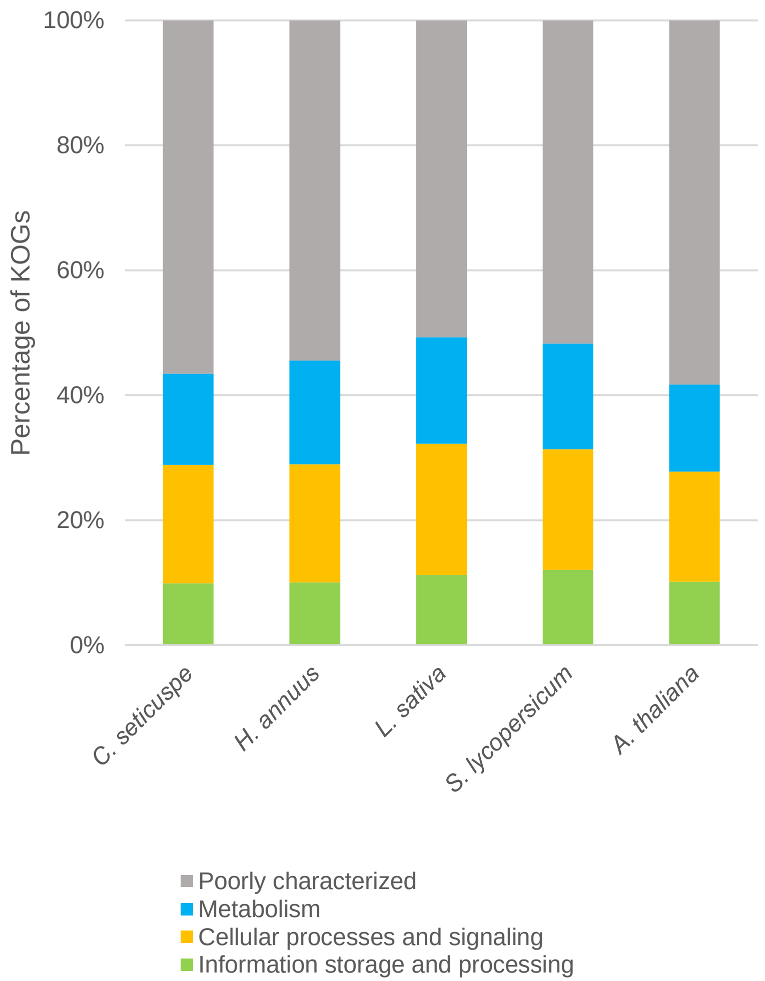

Supplementary Fig. S7. A: Numbers and ratios of genes annotated by the KOG database *in C. seticuspe* (CSE_r1.1_cds), sunflower (*H. annuus*, Ha412v1r1), lettuce (*L. sativa*, v5), tomato (*S. lycopersicum*, ITAG3.2) and *A. thaliana* (Araport11). **B:** Ratio of the classified KOG functional categories in annotated genes.

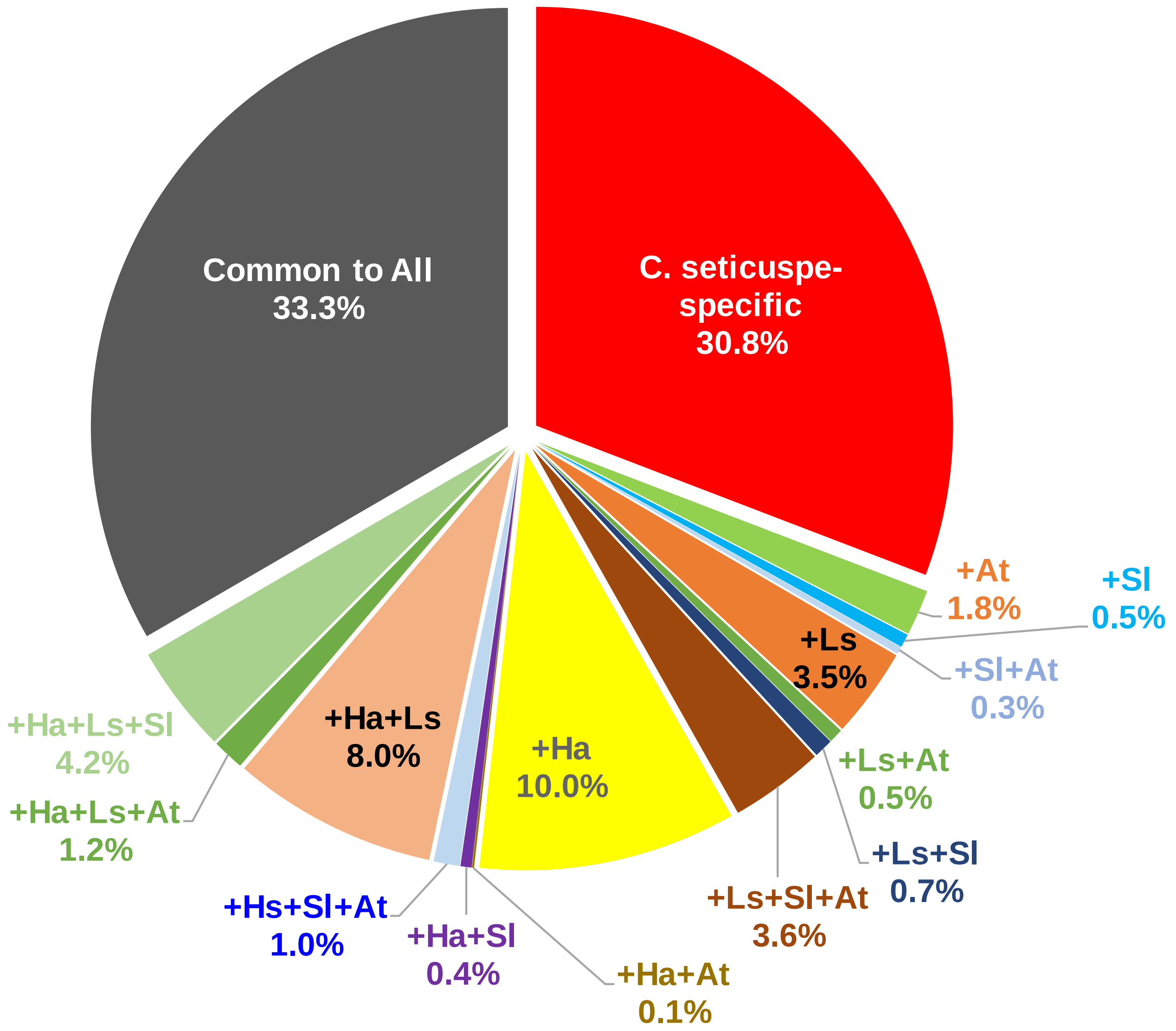

Supplementary Fig. S8. Ratios of genes of *C. seticuspe* (CSE_r1.1_cds) clustered with four other plant species. Ha, Ls, Sl and At represent sunflower (*H. annuus, Ha412v1r1_prot_v1.0*), lettuce (*L. sativa, Lsativa_467_v5*), tomato (*S. lycopersicum, ITAG3.2*) and *A. thaliana* (Araport11*)*, respectively.

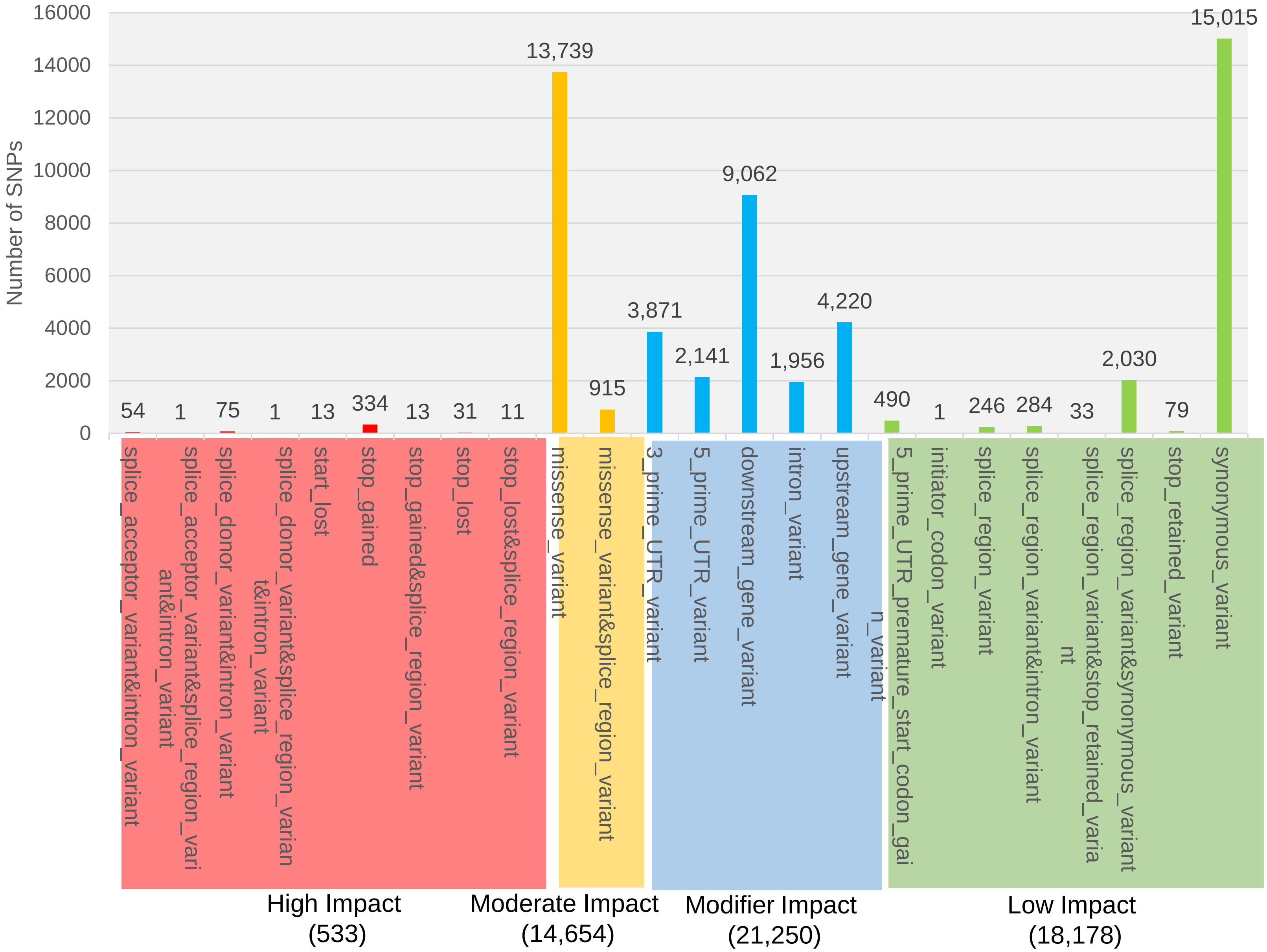

Supplementary Fig. S9. Numbers of genes identified as having annotated cultivated chrysanthemum SNPs by SnpEff. The numbers in parentheses indicate the total number for each category of impact. A total of 103,202 SNPs was mapped on the 22,470 genes.
